# Potential Therapeutic Use of PPAR*γ*-Programed Human Monocyte-Derived Dendritic Cells in Cancer Vaccination Therapy

**DOI:** 10.1155/2008/473804

**Published:** 2008-11-05

**Authors:** Adrienn Gyöngyösi, László Nagy

**Affiliations:** ^1^Department of Biochemistry and Molecular Biology, Medical and Health Science Center, University of Debrecen, 4010 Debrecen, Egyetem ter 1, Life Science Building, 4010 Debrecen, Hungary; ^2^Apoptosis and Genomics Research Group of the Hungarian Academy of Sciences, Research Center for Molecular Medicine, Medical and Health Science Center, University of Debrecen, 4010 Debrecen, Hungary

## Abstract

Dendritic cells (DCs) can regulate all elements of the immune system, and therefore are an ideal target for vaccination. During the last two decades, as a result of extensive research, DCs became the primary target of antitumor vaccination as well. A critical issue of antitumor vaccination is the phenotype of the dendritic cell used. It has been recently shown that several nuclear hormone receptors, and amongst them the lipid-activated nuclear receptor and peroxisome proliferator-activated receptor gamma (PPAR*γ*), have important roles in effecting the immunophenotype of human dendritic cells. It regulates primarily lipid metabolism and via this it influences the immunophenotype of DCs by altering lipid antigen uptake, presentation, and also other immune functions. In this review, we summarize the principles of antitumor vaccination strategies and present our hypothesis on how PPAR*γ*-regulated processes might be involved and could be exploited in the design of vaccination strategies.

## 1. DENDRITIC CELLS IN TUMORS

Dendritic cells (DCs) were discovered in mouse spleen by Ralph Steinman and Zanvil Cohn in 1973 [1]. Immature dendritic cells (IDCs) are sentinels of the immune system, continuously monitoring peripheral tissues for invaders, capture and process antigens, and migrate to the draining lymph nodes where they present peptides to naive Treg cells (T cells) and activate them [2]. The full activation of T cells requires special peptide-MHCI or peptide-MHCII complexes and additional signals from DCs in the form of various costimulatory molecules and cytokines. Furthermore, activated CD4^+^ T cells could be polarized to T helper 1 (Th1) and T helper 2 (Th2) subtypes. These processes are dependent on the cytokines interleukin-12 (IL-12), IL-4, and IL-10 secreted by mature DCs (MDCs). In response to IL-12, T cells polarize to Th1 and enhance CD8^+^ cytotoxic T-cell response against tumor cells or pathogens, while IL-4- and IL-10-activated Th2 cells promote humoral immune response and/or tolerance. Another important point is that DCs can induce T-cell tolerance to self-antigens and via this prevent and reduce autoimmune diseases.

Importantly, it appears that tumor tissues have characteristic immune environments with distinct DC subset distributions. Different DC subset localization within the compartments of tumor has been reported in colorectal cancer and oral squamous cell carcinoma patients. Dadabayev et al. investigated the infiltration pattern of DCs in human colorectal tumor samples analyzed with S100 and HLA class II DC markers. S100^+^ and CD1a^+^ DCs were found in tumor epithelium, in parallel with intraepithelial CD4^+^ or CD8^+^ T-cell infiltration and suggested increased disease-free survival, while HLA class II^+^ cells were observed in the stromal compartment, correlated with adversed outcome of the tumor [3]. Later they utilized CD208 (DC-LAMP) marker for marking MDCs and proved that CD208^+^ DCs were detectable in the peritumoral area, and infiltration of MDCs into tumor epithelium was correlated also with decreased patient survival [4]. In primary squamous cell carcinoma patients, IDCs and MDCs were characterized with distinct tissue localization patterns. Immature Langerhans cells (LCs) and DC-SIGN^+^ interstitial DCs were found inside the tumor tissue while the number of mature CD208^+^ DCs was limited. Moreover, CD123^+^ plasmacytoid DC representation in the tumor area was correlated with poor survival [5]. Importantly, DCs interact with tumor cells and cytokines produced by tumor cells or immune cells influence DC function and maturation. The tumor microenvironment affects DC differentiation from CD14^+^ monocytes and haematopoietic precursors promoting an early and dysfunctional maturation of DCs. Several reports described reduced the number of DC in peripherial blood, tumor tissues, and draining lymph nodes in cancer patients. Partial maturation of DCs by tumor-derived factors like IL-10, vascular endothelial growth factor (VEGF), and TGF-*β* induces self-tolarence and promotes conversion of naive T cells to regulator T cells, favoring development of suppressive T cells. Presence of tolerogenic T cells in tumor beds induces local immune suppression and alters the function of anticancer effector T cells. These cells, isolated from draining lymph nodes of patients with pancreas or breast cancer secrete IL-10 and TGF-*β*, prevent activated CD4^+^ CD25^−^ and CD8^+^ effector T cells, and suppress tumor-specific immune response [6]. Apart from the fact that DCs are involved in activation of Treg, there is an increasing amount of evidence about T cells, which can be recruited into tumors and affect DC development. Decreased CD80 and CD86 cell surface markers by T cells lead to reduced T-cell stimulatory ability of DCs [7, 8]. Immunosuppressive B7-3/4 molecules are upregulated on DCs upon DC-Treg interaction reserving a possible feedback loop to generate more regulatory T cells [9, 10]. Another immunosuppressive cycle is the conversion of DCs by Treg-secreted INF-*γ* and CTLA-4 into an indolamine 2, 3-dioxigenase (IDO) expressing cells which induce Treg generation and effector T-cell apoptosis [11, 12].

Classically, CD4^+^ T cells have been categorized into Th1, Th2, Treg, and Th17 subsets. However, TGF-*β* has a crucial role in Treg and Th17 cell development, the dichotomy of Treg/Th17 is dependent on IL6. Only a few pieces of evidence has been reported on the presence and regulation of Th17 cells by IL-2 in human cancer and experimental tumors. Muranski et al. reported that tumor-specific Th17 polarized cells mediated successful treatment of large established tumor in cutaneous melanoma-bearing mice. The therapeutic effect of the cells was dependent on their INF*γ* production [13].

Modulating factors released by the tumor environment cause defective functional maturation of DCs and affect the differentiation of immature myeloid-derived suppressing cells (MDSCs). The portion of MDSC is significantly increased in spleen, peripheral blood, and bone marrow of tumor-bearing patients and correlates with tumor progression [14–16].

In conclusion, DCs are one of the potent regulator cells in tumor development. The effects of DCs in cancer patients are contraversial: several reports demonstrated that myeloid-derived MDCs induce effective antitumor immune response and tumor regression. In spite of this, the suppressive tumor environment can alter the properties of DCs. The functional defects of DCs have an essentional role in cancer patient to impede succesful antitumor immune response. These tolerogenic DCs function as tumor-promoting cells. The future challenge of anticancer-based therapies is to overcome DC tolerogenecity and to reduce their negative effects in tumor progression.

## 2. DENDRITIC CELL-BASED CANCER THERAPY

**Figure 1:**
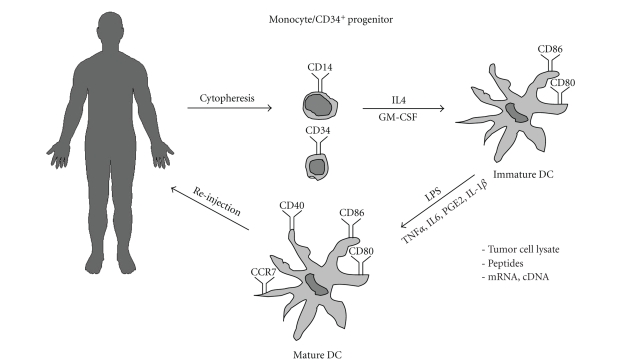
*General scheme of anticancer vaccination*. Dendritic cell progenitors (either CD34^+^ or CD14^+^ cells) are obtained using cytopheresis. Cells are differentiated using cytokines GM-CSF and IL-4. Immature dendritic cells are loaded with tumor lysate, peptides, or expression vector. DC maturation is induced and DCs are reinjected to patient.

It is clear that however in low number DCs are present in tumors and can be used to elicit antitumor immune response. The challenge and goal of anticancer therapy is to elicit an effective cellular immune response against tumor cells and evoke clinical response in treated patients with negligible side effects. The discovery of isolation techniques and methods for differentiating DCs in vitro gave us the possibility to generate DCs that could be loaded by tumor-specific antigens or peptides. In this therapeutic approach, one can define DC-vaccine as a DC loaded with tumor-specific antigen. The first DC-based clinical trial against B-cell lymphoma was reported by Hsu et al. [17]. One important question in DC vaccination is to decide whether to use an ex vivo or in vivo vaccination strategy. The ex vivoapproaches (see Figure 1) allow to monitor the quality of the cells during the differentiation procedure, analyze cell surface markers, the proper maturation state, cell viability, or subtype specificity of DCs by FACS analysis. It is also possible to evaluate the effective tumor antigen-specific T-cell response by ELISpot, mixed leukocyte reaction (MLR) before targeted DCs are introduced back to the patient. The possible sources of human DCs are CD34^+^ precursors, hematopoetic progenitors, and monocytes, isolated from blood by cytopheresis, adherent techniques or magnetic-based immunoselection, or immunodepletion [18–20]. IDCs can be differentiated from peripheral blood-derived monocytes in vitro in the presence of GM-CSF and IL-4 [18, 20]. Alternatively, DC precursors can be isolated from human peripheral blood, but for effective anticancer therapy one has to obtain high amount of targetable DCs. In a clinical trial, FLT-3 ligand-expanded DCs were prepared from the blood of colon and nonsmall cells of lung cancer patients. Because of the limited blood DC number, patients underwent FLT-3 treatment before DC isolation. As a result, three times more PBMC was obtained from these patients after standardized leukopheresis as compared to control patients. The isolated patient-derived DCs showed immature CD83^−^/CD40^low^/CD80^low^/CD86^low^ phenotype, but after two days in culture, cells started to express CD83, elevated levels of CD86 and CCR7 proteins, which reflect MDC phenotype and migration capacity [21]. DC-vaccine studies utilize DCs loaded with peptide fragments or whole proteins providing an opportunity to present all potential peptide sequences of the antigen to recognize even more specific T-cell clones and tumor lysates exogenously [22]. Alternatively, one can target DCs endogenously with antigen-coded mRNA or cDNA [23]. After loading IDCs with tumor-specific antigens, it is very important to add adequate maturation agents (e.g., proinflammatory cytokines, LPS, CD40L) to ensure that DCs achieve their maximum migratory capacity to the lymph nodes, otherwise only a small portion of antigen-loaded DCs could migrate to the site of the naive T-cell activation. Following quality control steps, the generated DC vaccines have to be reinjected into the patients. An important issue is also the DC injection site. DC vaccines could be reinjected to patients by intravenous, subcutaneous, intradermal, or intralymphatic injections. In a clinical study, the efficiency of different injection sites was compared by Fong et al. [24]. According to their results, the intradermal or intralymphatic administration was more effective compared to intravenous injection. In general, DC delivery via the skin is preferable to intravenous injection. Combining the different routes of reinjections may be beneficial, depending on the tumor localization. Most of the early phase I clinical trials, using the ex vivo approach, have not shown long-term tumor regression or improved survival. Probably it is mostly due to the fact that in these studies the researchers selected only advanced-stage cancer patients who were immonosuppressed by recurrent tumors or by chemotherapy. However, an in vitro approach could provoke Th1 cell response in metastatic malignant melanoma [25].

As far as the migratory capacity of DCs is concerned, less than 5% of the MDCs reach the lymph nodes after intradermic injection [26]. Therefore, it would be more beneficial to activate and target DCs within the host. In this case, we are not concerned with cell isolation or differentiation protocols, but rather DC-initiated tumor-specific immune responses have to be monitored inside the body. Monoclonal antibodies and fusion construct can be used for more productive tumor antigen delivery directly into the DCs and probably the cells do not need to be cultured in vitro. Many vaccination studies target DC-specific c-type lectin receptors for efficient targeting of tumor antigens into the cells. These receptors bind to particular self- or nonself-sugar patterns by means of their carbohydrate recognition domain (CRD) and have roles in endocytotic antigen uptake [27]. One of them, DEC205/DC205, is expressed at high levels by MDC in mice, but human B cells, NK cells, monocytes, and macrophages also express this receptor [28]. Bozzacco et al. designed a fusion monoclonal antibody construct by taking the light and heavy chain coding cDNA sequence of an anti-DEC205 antibody and by inserting different gag p24 peptides at the carboxy terminus of the heavy chain. According to their results, these antibodies increased antigen presentation in the treated HIV-infected patients. They could further demonstrate DC-primed cross-presentation of internalized, nonreplicating proteins to MHCI complexes inducing CD8^+^ T-cell activity [29]. These results support the feasibility of engineering tumor-specific peptide fragment into DC-targeted antibodies against various types of cancer in vivo.

## 3. THE ROLE OF THE PPAR*γ* RECEPTOR IN DENDRITIC CELLS

### 3.1. PPAR*γ*-altered phenotype of DCs

Nuclear hormone receptors are ligand-activated transcription factors. There are three different PPAR isoformes in the human body and these show distinct tissue-specific distribution with different physiological functions. PPAR*α* is most highly expressed in the liver, skeletal muscle, kidney, and heart, and it regulates fatty acid oxidation [30]. PPAR*δ* shows a ubiquiter distribution while PPAR*γ* expression can be detected in various cell types like adipocytes, macrophages, and DCs. The receptor was initially described in mouse adipose tissue [31] and its role in myeloid development was shown by Nagy and Tontonoz in 1998 [32, 33]. PPAR*γ* knockout mice are lipodystrophic and die of placental defect, showing the essential regulatory role for the receptors in embryonic differentiation [34]. Moreover, high level of PPAR*γ* expression can be detected in monocyte-derived macrophages in atheroscleric lesions [33]. PPAR receptors heterodimerize with retinoid X receptors (RXRs) in the nucleus and bind to certain receptor-specific response elements (PPREs) in the promoter or enhancer regions of their target genes [35]. The PPRE contains direct repeat sequences separated by one base pair (DR1). Endogenous or exogenous ligands bind into the ligand-binding domain (LBD) of PPAR*γ* and modulate PPAR*γ*-mediated gene expression. PPAR*γ* can be activated by components of the oxidized low-density lipoprotein (oxLDL) and prostaglandin derivate (e.g.,15d-PGJ2) [32, 36]. Ligand activation of the receptor induces the expression of CD36 scavenger receptor which in turn leads to oxLDL uptake of macrophages and this metabolic process can lead to foam cell formation [32]. From these studies, we know that PPAR*γ* regulates fatty acid uptake into the cell by induced cell surface receptors and it also promotes lipid storage and accumulation. Beside this fundamental regulatory role in metabolism, the receptor also functions as a key modulatory factor in macrophage immune function [37]. Earlier microarray data suggested that the PPAR*γ* gene is upregulated during monocyte-to-DC differentiation [38]. According to our experiments and those of others, the receptor is immediately upregulated in cultured DCs, while it is barely detectable in monocytes [39]. We have shown that the transcription factor in this system is active, because synthetic agonists induce dose-dependent gene expression of the bone fide PPAR*γ* target gene FABP4 in IDCs [39, 40]. Through global gene expression analysis, we found that PPAR*γ*-activated genes involved primarily in the first 6 hours are involved primarly in lipid metabolism and transport (CD36, LXR*α*, and PGAR). Genes, coupled to the immune regulatory role of human DCs, were upregulated only for 24 and 120 hours after ligand treatment. It is possible that immunophenotype of DCs could be altered by PPAR*γ* activation indirectly through activation of lipid metabolism and signaling pathways [41].

In terms of DC-based vaccination therapy, the most important question is how PPAR*γ* activation might effect the DC-initiated immune responses and DC phenotype. PPAR*γ* expression was first detected in murine DCs by Faveeuw et al. and they reported that there is a PPAR*γ* receptor-dependent inhibition of IL-12 secretion of IDCs and MDCs [42]. Furthermore, it was also shown that PPAR*γ* ligand activation caused anti-inflammatory cytokine production in macrophages [37]. These findings support the idea that PPAR*γ* might have an essential role in the APC-based DC-vaccine therapies. The DC-secreted IL-12 is indispensable for Th1 cell promotion and CD8^+^ T-cell activation. Earlier publications by Gosett et al. and Nencioni et al. assessed that PPAR*γ* ligand activation alters the immunogenicity of human monocyte-derived DCs [43, 44]. During DC maturation, costimulatory molecules (CD40, CD80, and CD86) are upregulated on the surface of DCs [2]. Some bacterial products, such as LPS, are able to induce signals via TLR receptors or CD40 molecules induce IL-12 secretion of DCs. They have also found that upon ligand activation of PPAR*γ*, the phenotype and cytokine expression patterns of the cells were changed [43]. PPAR*γ* ligands altered iDC-specific surface markers involved in APC function. The CD83 activation marker expression in treated MDCs was uneffected, which means that PPAR*γ* ligand-activated cells showed mature phenotype. After ligand activation, elevated CD86 protein level was detected on the surface of MDCs. They also showed that activation of PPAR*γ* inhibits the secretion of IL-12p70 active form into the supernatant by MDCs while the levels of IL-6 and IL-10 were unchanged. Furthermore, chemokines involved in Th1 cell recruitment (IP-10, RANTES, and MIP1*α*), were also decreased after ligand treatment in the same study. Nencioni et al. later characterized the effects of PPAR*γ* on DC maturation and found that ligand activation reduced the surface expression of CD1a molecule in a concentration-dependent manner, resulting in an unusual phenotype of differentiated IDCs [44]. Lower levels of IL-10, IL-6, and TNF*α* cytokines were measured upon ligand treatment. PPAR*γ* agonist impaired the allogenic T-cell stimulating capacity in MLR assays and the secreted INF*γ* concentration was also reduced. T-cell activation capacity could not be restored by IL-12 administration suggesting that the impaired T-cell activation of MDCs was not only due to lack of IL-12 expression but also to other effects that modulate DC maturation process were involved.

In conclusion, the PPAR*γ* ligand-activated cells not only impede the naive T cell to Th1 cell differentiation, but these cells also showed decreased antigen-specific T-cell response. Appel et al. reported that important anti-inflammatory effects of the receptor as ligand activation of the PPAR*γ* receptors inhibited the LPS-activated MAP kinase and NF-*κ*B proinflammatory signaling pathways, probably due to transrepression mechanism in DCs that subvert IL-12 expression [45].

Flow cytometry measurements performed by some of us largely supported the phenotypic results reviewed above [39]. Furthermore, when treated DCs with PPAR*γ*, we detected that enhanced endocytosis in the form of enhanced latex bead uptake and ligand-treated cells were CD1a^−^. We could not detect any differences in case of HLA-ABC molecule expression, suggesting that the MHCI-mediated peptide antigen presentation capacity of the cell is probably not affected [46]. As reported by Angeli, PPAR*γ* inhibits the expression of CCR7 on the surface of MDCs and this decreased the migration of DCs in mice. In this model, TNF*α*-induced epidermal LC motility from epidermis to dermal lymph nodes was reduced by PPAR*γ* ligand treatment. They also found that ligand-activated PPAR*γ* impaired the steady-state migration of DCs from the mucosal to the thoracic lymph nodes, but the maximal inhibitory effect was detected at a considerably high concentration of the PPAR*γ* agonist rosiglitazone (10 *μ*M) that suggested receptor-independent effects [47].

Summarizing these data, PPAR*γ* activation in DCs prevented IL-12 secretion, lowered CD80/CD86 ratio, and probably shifted naive T-cell differentiation toward Th2 cells. According to our own experiments, PPAR*γ* agonist rosiglitazone at 2.5 *μ*M concentration did not decrease the activation of allogeneic T cell and INF*γ* production [39]. So far, no one was able to detect Th2 response in MLR in response to PPAR*γ* ligand activation.

### 3.2. The role of PPAR*γ* in CD1d-mediated lipid antigen presentation

**Figure 2:**
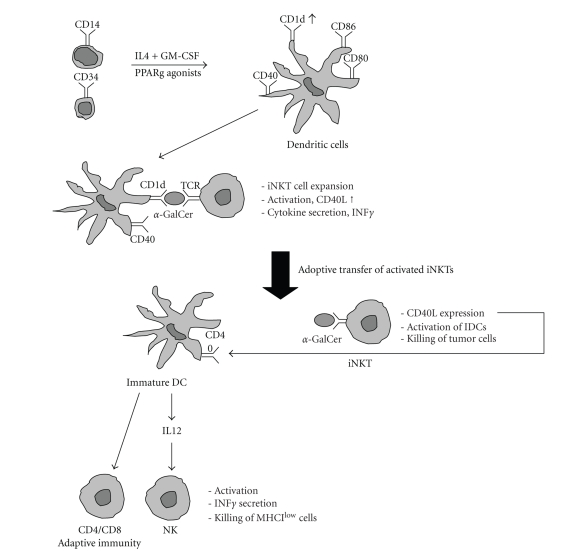
*The molecular basis for the potential use of PPAR*γ*-programed dendritic cells during tumor vaccination.* DC progenitors are differentiated in the presence of PPAR*γ* agonists. A PPAR*γ*-programed DC showed increased CD1d expression. In the presence of *α*GalCer, the treated DC is capable of inducing iNKT cell expansion. The adoptively transfered iNKTs can induce activation of iDCs and IL-12 secretion in cancer patients. This can lead to improved ability to kill tumor cells.

Szatmari et al. provided evidence that PPAR*γ* activation could effect the lipid antigen presentation capacity of monocyte-derived DCs through upregulated expression of CD1d molecule on the surface of DCs [39]. This finding links PPAR*γ* to invariant natural killer T (iNKT) cells. After isolation, monocytes fail to express CD1 group I molecules (CD1a, b, c) but the CD1a protein is upregulated during monocyte-to-IDC differentiation [20]. Inversely, the CD1 group II molecule CD1d is expressed at high levels on monocytes and downregulated on the surface of DCs [39]. Induced signaling pathways are able to regulate CD1d gene expression and lipid metabolism upon PPAR*γ* ligand treatment [41]. Utilizing PPAR*γ* agonist treatment, Gogolak et al. could induce the expression of CD1d molecules along with downregulation of CD1a at both mRNA and protein levels [48]. Later we established that PPAR*γ* ligand activation enhanced indirectly the CD1d expression by turning on endogenous lipophilic ligand synthesis in the DCs through activation of the expression of retinol dehydrogenase 10 (RDH10) and retinaldehyde dehydrogenase type 2 (RALDH2) enzymes, which are involved in retinol and retinal metabolism and endogenous all-trans retinoic acid (ATRA) production from retinol [46]. The intracellularly synthesized ATRA induced CD1d and other retinoic acid receptor alpha (RAR*α*) target genes in DCs. We looked at the functional role of PPAR*γ*-induced retinoid-regulated CD1d expression on DC surface. DCs pulsed with synthetic alpha-galactosilceramide (*α*GalCer) ligand for 24 hours elevated iNKT cell expansion and INF*γ* secretion [39, 41]. As CD1d-mediated lipid antigen presentation is essential for iNKT cell activation, we could conclude that PPAR*γ*-induced CD1d expression can be translated to the increased activation and proliferation of iNKT cells under these in vitro conditions [39]. Our results suggest that combination of PPAR*γ* activator ligands along with *α*GalCer during the differentiation of DCs might be beneficial in iNKT-based adoptive transfer therapy (see Figure 2).

## 4. CD1d-RESTRICTED iNKT CELLS IN CANCER THERAPY

### 4.1. iNKT cell-based anticancer effects in animal models

Besides DCs and T cells, there are other important cell types contributing to antitumor immunity. iNKT cells are a unique T-lymphocyte population. These cells share both NK (CD161) and T-cell-specific markers (TCRs) on their surfaces. iNKT cells have restricted T-cell receptors (TCRs): in mice, the most frequently expressed *α*-chain rearrangement is V*α*14-J*α*18 while human NKT cells express V*α*24-J*α*18/V*β*11 TCRs (reviewed by Godfrey and Kronenberg [49]). For iNKT activation, it is essential to interact with cells displaying the evolutionarily conserved CD1d, nonclassical antigen-presenting molecules that present glicolipids in the context of hydrophobic antigen binding to these cells [50, 51]. *α*GalCer is the most frequently used lipid ligand for iNKT activation. It is derived from a marine sponge.

*α*GalCer has shown antimetastatic activity in various experimental tumor models (e.g., B16 melanoma, Lewis lung carcinoma, FBL-3 erytroleukemia, Colon26, and RMA-S 3LL tumor cells) in vivo [52–54]. This effect of the compound was tested in CD1d^−/−^, Ja281^−/−^ RAG^−/−^ NKT mice, which have no iNKT cells and in NK-depleted wild-type mice. The results indicated that the antitumor effect of the glycolipid was abolished on all of the three tested genetic backgrounds in mice and the *α*GalCer-mediated antimetastatic function likely acts through iNKT cell activation and NK-like effector function [52]. Adoptive transfer experiments provided further proof for the key role of iNKT cell-secreted INF*γ* in the antimetastatic role of *α*GalCer in mice. Furthermore, activation and proliferation of NK cells downstream to iNKT activation, and subsequent INF*γ* production was also required to be essential for antimetastatic cytotoxic activity in vitro and in vivo [55, 56]. *α*GalCer activates iNKT cells, which produce INF*γ*, and secondary activates NK cells. These activated NK cells have been implicated also in the regulation of angiogenesis during tumor development. The *α*GalCer treatment inhibits the subcutaneous tumor growth, tumor-induced angiogenesis, and epithelial cell proliferation, which are required for tumor vessel formation [57]. Later it has been established that *α*GalCer treatment, in combination with IL-21, prolonged and elevated the NK cell cytotoxicity, maturing NK cells into perforine-expressing cells by IL-21. Moreover, this combination inhibited spontaneous tumor metastases. Presentation of *α*GalCer by DC to iNKT cells in contrast to soluble compound injection was even more effective in the suppression of metastasis formation [58]. Similar successful antitumor effects of *α*GalCer-pulsed DC have been published by Toura et al. using B16 melanoma liver metastasis and lung metastasis of LLC model in vivo. Beside the inhibition of metastatic nodule formation in these tissues, *α*GalCer-DC administration also has a significant beneficial effect in the regression of established nodules [59, 60].

### 4.2. iNKT cells in human cancer therapy

Human V*α*24^+^ iNKT cells also mediate *α*GalCer-dependent antitumor activity by perforin-dependent cytotoxic lysis against Daudi lymphoma and other various cell lines [53, 61]. Others demonstrated effective direct iNKT-mediated cytotoxicity only against CD1d^+^ cell lines such as U937, while CD1d^−^ cell lines were killed only after CD1d transfection into the cells. NKT cells provoked NK cell-induced cytotoxitity by IL-2 and INF*γ* secretion [62].

The crosstalk between innate and adaptive immunity was established in several anticancer studies. This linkage could be mediated via reciprocal interaction between iNKT cells and iDCs. Upon *α*GalCer activation, iNKT cells express CD40L [63]. The CD40 ligand binds to the CD40 molecules of DCs and triggers IL-12 expression and secretion by the DCs. The produced IL-12 generates a positive feedback and induces INF*γ* secretion by the iNKTs [63, 64]. The secondary activation of DCs leads to NK, CD4^+^, and CD8^+^ T-cell activation by standard activation of memory T cells and adaptive immune response against peptides presented by DCs [65–67].

The fact that *α*GalCer-loaded DC could trigger iNKT expansion and mediate antitumor immune response in several in vitro experiments and in vivo antimetastatic models in mice supported the notion that using *α*GalCer-pulsed DCs for iNKT activation in cancer patients in situ might induce an effective anticancer therapy. Other alternative approach could be the adoptive transfer of in vitro expanded and activated iNKT cells to patients.

The number of NKT cells in cancer patients is significantly lower compared to healthy volunteers. Giaccone et al. showed the disappearance of iNKT cells after 24 hours of *α*GalCer administration from the peripheral blood of patients and only transient iNKT activation was registered in some individuals [68]. Others found quantitative defects in iNKT cell-derived INF*γ* production among patients with advanced prostate cancer [69]. Showing that NK cells were able to respond to IL-12, those cells could secrete increased levels of INF*γ* demonstrating the selective loss of INF*γ*-secreting capacity of iNKT cells in patients [69]. As it was expected from mouse experiments, Chang et al. were able to expand the number of iNKT cells for more than a month in all treated patients, proving that *α*GalCer-pulsed MDCs could be more effective than using *α*GalCer-pulsed IDC or the soluble compound [70]. However, the levels of IL-12p40 and IL-10 in the serum were elevated after the treatment and iNKT cells showed reduced-INF*γ* secretion. The future challenge of this type of tumor therapy is to induce extended iNKT number and activity. One possible approach is to use additional pharmacological ligands upon cancer therapies. It has been shown that the thalidomide analogue, lenalidomide (LEN), enhanced the predominant iNKT cell expansion in vitro and in vivo in response to *α*GalCer-loaded DC. LEN elicits higher level of INF*γ* secretion in response to *α*GalCer-loaded DC, suggesting that LEN might be restoring the INF*γ*-producing activity of iNKT cells in cancer patients [71]. Alternative possibility is the adoptive transfer method: in phase I clinical trial, adoptive transferred iNKTs were used in patients with malignancy to increase the number of iNKT cells [72]. They expanded iNKT cells in vitro in the presence of IL-2 and *α*GalCer. The activated iNKT cells showed cytotoxic activity against PC-13 and Daudi human cancer cell lines. Reinjection of activated iNKT cell into the patients enhanced the level of INF*γ* secreting iNKT cells in the peripheral blood from day one up to two weeks [72].

## 5. DENDRITIC CELL PPAR*γ* IN ANTICANCER THERAPY

Despite the enormous research effort, cancer is still a significant clinical problem as well as basic science problem. However, immunotherapy opened up some possibility in the fight against cancer. General APC features of DCs are capturing antigens in the periphery, processing, and enhancing MHC-peptide complex presentation capacity to naive T cells. These phenomena highlighted possible roles for DCs in anticancer therapy. However, DC-based vaccination often does not elicit clinical responses and fails to ensure long-time tumor regression in patients with malignant tumors. One reason for this failure could be the immunosuppressed state of the patient, for example, due to chemotherapy in the therapeutical history. Recently, we have identified a new target gene, ABCG2, which is transcriptionally regulated by PPAR*γ* in DCs. ABCG2 transporters modify the drug resistance against anticancer agent of PPAR*γ* agonist-treated DCs. PPAR*γ* has a protective function in these cells, and using PPAR*γ*-specific ligands during in vitro differentiation could revert the xenobiotics-induced toxicity in DCs [73].

Due to the fact that we did not find reduced capacity of DCs to activate T cell in MLR assays, we concluded that ligand activation does not suppress DC-mediated peptide antigen presentation. For effective anticancer therapy, one should provoke adaptive immune response against tumor-specific peptides presented by DCs. In mouse experiments, simultaneously added *α*GalCer and peptide-loaded DCs induced CD4/CD8 T-cell-specific anticancer immune response mediated by iNKT cell. In case of human patients, *α*GalCer-loaded DCs could not induce adaptive immunity, partly because of the ineffective INF*γ* secretion by iNKT cells. Pharmacological approaches like LEN may solve this problem. The ability of PPAR*γ* to upregulate CD1d expression on DCs raises the possibility to use receptor agonists in iNKT-based adoptive transfer treatments.

Many features of DCs, which are critical during DC-vaccination design, are affected by PPAR*γ*. Reduced migratory capacity, inhibited IL-12 cytokine production, inadequate Th1 and CD8^+^ T-cell response, and presumed generation of IL-10-producing tolerogenic DC could influence the outcome of DC-based vaccination therapies against cancer. Based mainly on these in vitro results, activation of the PPAR*γ* receptor in tumor peptide-pulsed DCs could be less beneficial in terms of in vivo vaccination strategies. At the same time, increased phagocytic capacity, increased CD1d expression, and iNKT activation potential are useful features of PPAR*γ*-programed DCs. In spite of the vast amount of in vitro obtained results on the potential role of PPAR*γ* in DCs, the most controversial issue remains open: whether synthetic PPAR*γ* agonists have significant modifying effects on antitumor immune response in vivo or not. Further in vivo studies are needed to clarify the receptor-specific immunomodulatory effects of PPAR*γ* ligands (agonists or antagonists) in cancer patients. For that, the use of siRNA-based gene-silencing techniques or DC-specific PPAR*γ*, KO animal models would probably be useful.
